# Imprinted MicroRNA Gene Clusters in the Evolution, Development, and Functions of Mammalian Placenta

**DOI:** 10.3389/fgene.2018.00706

**Published:** 2019-01-18

**Authors:** E. Cécile Malnou, David Umlauf, Maïlys Mouysset, Jérôme Cavaillé

**Affiliations:** ^1^Centre de Physiopathologie de Toulouse Purpan, Université de Toulouse, CNRS, INSERM, UPS, Toulouse, France; ^2^Laboratoire de Biologie Moléculaire Eucaryote, Centre de Biologie Intégrative, CNRS, UPS, Université de Toulouse, Toulouse, France

**Keywords:** genomic imprinting, microRNA, post-transcription control, placenta, mammals

## Abstract

In mammals, the expression of a subset of microRNA (miRNA) genes is governed by genomic imprinting, an epigenetic mechanism that confers monoallelic expression in a parent-of-origin manner. Three evolutionarily distinct genomic intervals contain the vast majority of imprinted miRNA genes: the rodent-specific, paternally expressed C2MC located in intron 10 of the *Sfmbt2* gene, the primate-specific, paternally expressed C19MC positioned at human Chr.19q13.4 and the eutherian-specific, maternally expressed miRNAs embedded within the imprinted Dlk1-Dio3 domains at human 14q32 (also named C14MC in humans). Interestingly, these imprinted miRNA genes form large clusters composed of many related gene copies that are co-expressed with a marked, or even exclusive, localization in the placenta. Here, we summarize our knowledge on the evolutionary, molecular, and physiological relevance of these epigenetically-regulated, recently-evolved miRNAs, by focusing on their roles in placentation and possibly also in pregnancy diseases (e.g., preeclampsia, intrauterine growth restriction, preterm birth).

## Introduction

**Figure 1:**
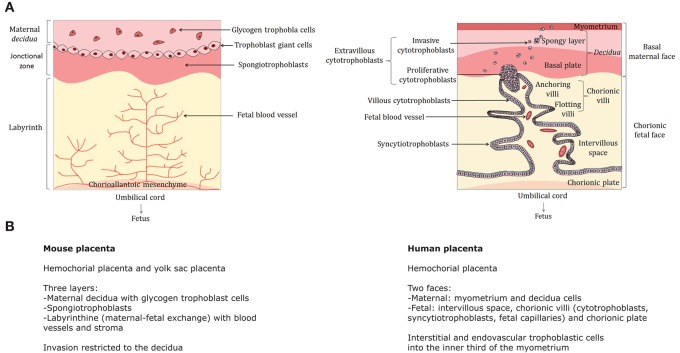
Comparative structure of the human and mouse placentas—**(A)** The cellular organization of the mouse (left) and human (right) is compared. The human placenta is a discoid hemochorial organ composed of two faces: the basal maternal face in contact with the endometrium and the chorionic fetal face into which the umbilical cord is implanted in connection with the fetus. The maternal part includes the basal *decidua* (or *decidua*), itself composed of a deep spongy layer attached to the myometrium and the basal plate consisting mainly of decidual cells. The fetal part is composed by the intervillous space, where the chorionic villi are located, and the chorionic plate. The chorion is composed by 15–30 cotyledons, each consisting of chorionic villi where the exchanges between mother and fetus take place. Each villus comprises a floating part (floating villi) in the intervillous space, which is the exchange zone with maternal blood, as well as a part attached to the maternal *decidua*, called anchoring villi. The flotting villi consists of a stromal center including fetal blood vessels, macrophages (also called Hofbauer cells) and fibroblasts. The stroma is surrounded by a basement membrane covered by two major cell populations: cytotrophoblasts (mononuclear cells) and syncytiotrophoblasts (plurinucleated cells), both derived from trophoblasts. Cytotrophoblasts are located in the inner layer of chorionic villi and syncytiotrophoblasts are external. They are formed and renewed throughout pregnancy by fusion of cytotrophoblasts. The anchoring villi consists of columns of extravillous cytotrophoblasts that are first proliferative (proliferative cytotrophoblasts) and then migrate to the internal third of the myometrium (invasive cytotrophoblasts). For further details: (Dilworth and Sibley, 2013; Schmidt et al., 2015). **(A)** was created using Servier Medical Art, licensed under the Creative Commons Attribution 3.0 Unsupported License. **(B)** Main anatomical and cellular features of the mouse and human placentas. Murine placenta harbors significant differences with respect to the primate placenta. It is composed of three layers: the maternal *decidua* with glycogen trophoblast cells, a junctional zone with giant spongiotrophoblasts and the labyrinth (where materno-fetal exchanges take place). Invasion is restricted to the *decidua*.

The placenta is a transient organ that develops at the interface between maternal and fetal tissues and orchestrates diverse physiological functions during pregnancy. The placenta provides nutrient and oxygen supply and it also ensures waste elimination, protection against infections and hormone synthesis (Burton and Fowden, 2015). Strikingly, it is one of the most variable organs in terms of shape, structure and cell types, as illustrated in Figure 1, where the cellular organization of the human and mouse placental tissues is compared. Of note, placental-like structures can also be found in non-placental mammals, as well as in other taxa such as reptiles, fishes or even invertebrates (Carter, 2012; Roberts et al., 2016; Griffith and Wagner, 2017). Although the mechanisms whereby such an invading and multifunctional organ has evolved within each lineage are not well understood, several major driving forces are likely involved in the evolution and diversification of the placental tissues in mammals. These include duplication events followed by gene diversification, rewiring of pre-existing gene regulatory networks, exaptation of endogenous retroviruses, ecological pressures, evolutionary conflict between mother and fetus and genomic imprinting (Frost and Moore, 2010; Kaneko-Ishino and Ishino, 2010; Carter, 2012, 2018; Chuong et al., 2013; Lavialle et al., 2013; Renfree et al., 2013).

**Figure 2:**
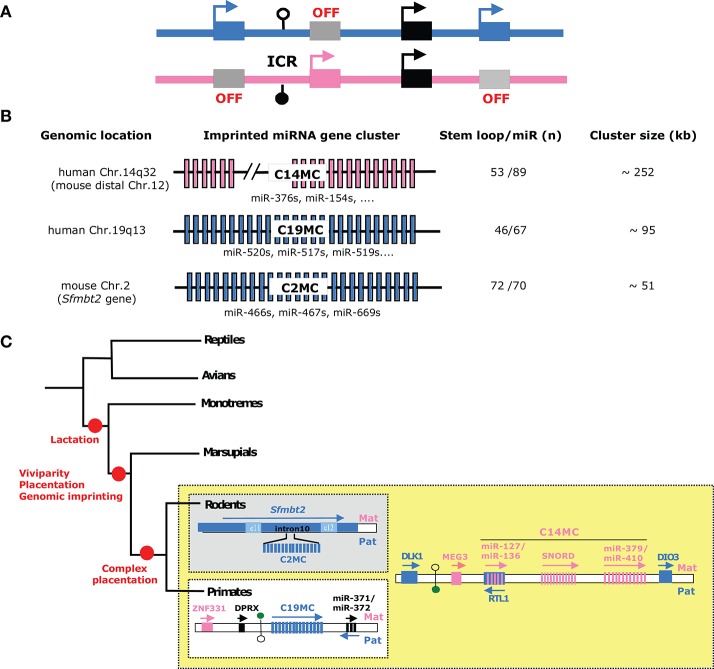
Imprinted microRNA gene clusters—**(A)** Schematic representation of a generic imprinted gene cluster. In eutherian species, most imprinted genes do not locate randomly along chromosomes but are grouped as large imprinted gene clusters (~80–3,700 kb in size) that can host both maternally and paternally expressed genes (*n* ~3–12). Each imprinted gene cluster contains a germ-line derived, differentially methylated region, also referred to as imprinting center region (ICR). Through diverse mechanisms (e.g., enhancer-blocker, promoter for long non-coding RNA, higher-order chromatin structure…), ICRs orchestrate imprinted expression *in cis* and in a domain-wide manner. For further details: (Barlow and Bartolomei, 2014). Paternally and maternally expressed alleles are denoted as, respectively, blue and pink rectangles and epigenetically silent allele are colored in gray. Note that some genes can escape genomic imprinting control (black rectangles). DNA methylation profiles are symbolized by filled and open lollipops (methylated and unmethylated, respectively). ICRs are also marked by allele-specific post-translational histone modifications (not shown). **(B)** Schematic representation of the three known imprinted miRNA genes clusters: C14MC, C19MC, and C2MC (also known as *Sfmbt2* cluster). The number of hairpin (pre-miR) and mature miRNA species is given (based on miRbase 22 release) (http://www.mirbase.org/). Note that some miRNAs may not fulfill all criteria to be considered as *bona fide* miRNA. Some representative miRNA families are also indicated. **(C)** Imprinted miRNA gene clusters represent gene innovation during the course of mammalian evolution. The simplified phylogenetic tree highlights the acquisition of genomic imprinting and several major features of mammalian species (red circles). C14MC is conserved in placental (eutherian) mammals while C19MC and C2MC are only found in primates and rodents, respectively. Note that C19MC miRNAs can be found in Old and New world monkeys, but apparently not in Tarsiers, nor in prosimians (lemurs, bushbabies and Lorises). To the best of our knowledge, imprinted (paternal) expression at C19MC was only experimentally demonstrated in human placenta. The drawing is not to scale.

Genomic imprinting is an epigenetic mechanism whereby the expression of an allele depends on which parent it was transmitted from (Barlow and Bartolomei, 2014). In other words, some imprinted genes are only expressed from their maternal allele, while others are only expressed from their paternal allele. This therefore implies that imprinted genes are functionally haploid. Imprinted genes play essential roles in pre- and postnatal growth as well as in behavior and metabolic adaptation in the perinatal period and adulthood (Cleaton et al., 2014). In eutherians, ~150 autosomal genes are subject to this outstanding epigenetic regulation, with many of them belonging to large chromosomal domains (Figure 2A). Several lines of evidence have strengthened the link between genomic imprinting and complex placentation (Renfree et al., 2009). Indeed, only a few imprinted genes were discovered in marsupials and none have been detected in the egg-laying monotreme mammals. Acquisition of genomic imprinting occurred after the divergence of monotremes from marsupials and eutherians and therefore coincides with more invasive placentation and increased gestational length (Renfree et al., 2013). Even more tellingly, many imprinted genes are predominantly expressed in the placenta—or even imprinted solely in this organ—where they influence fetal and placental growth as well as nutritional resource acquisition (Frost and Moore, 2010; Fowden et al., 2011; Varmuza and Miri, 2015). Finally, alterations in the dosage of some human imprinted genes are linked to several pregnancy-related diseases (e.g., pre-eclampsia, intra-uterine growth retardation) as well as with disorders caused, at least in part, by placental dysfunctions (Peters, 2014; Monk, 2015). In eutherians, genomic imprinting confers mono-allelic expression to a great variety of genomic loci including protein-coding genes but also intronless retrogenes, retrotransposon-related genes, long non-coding RNA (lncRNA) genes as well as C/D box small nucleolar RNA (SNORD) or microRNA (miRNA) genes.

miRNAs are tiny, single-stranded RNAs (~19–21 nt) that are processed from imperfect stem-loop structures embedded within larger primary transcripts (pri-miRNAs). Most of them function as antisense RNAs, by forming short base-pairing to partially complementary sequences often positioned in the 3′ untranslated regions (3′-UTRs) of mRNAs (Bartel, 2018). The binding of a miRNA and its associated Argonaute proteins onto the 3′-UTR modulates the post-transcriptional fate of the targeted mRNA, either by limiting its translation and/or by accelerating its decay (Jonas and Izaurralde, 2015). The impact of a miRNA on the repression of its target is often relatively mild and we are far from fully apprehending all the features that govern the specificity of such short, imperfect miRNA::mRNA interactions *in vivo*. It is, however, well known that perfect base-paring of nucleotide at positions 2–7 from the 5′-end of the miRNA (the “seed region”) is necessary, and sometimes sufficient, to elicit post-transcriptional gene silencing (Lewis et al., 2005). However, after more than two decades of intense research, the identification of physiologically relevant mRNA targets remains still challenging. Indeed, the minimalist requirement for base-pairing implies that each miRNA could theoretically target a huge number of protein-coding transcripts (*n*~100–1,000). Reciprocally, a given mRNA could also be theoretically targeted by numerous miRNAs. In any event, miRNAs are now recognized as pivotal posttranscriptional regulators in a broad range of biological processes, particularly in response to developmental, metabolic, physiological, or environmental challenges (Bartel, 2018). Accordingly, mammalian miRNAs exert regulatory roles in many aspects of placental development and functions, from trophoblast invasion to immunity through angiogenesis (Doridot et al., 2013; Sadovsky et al., 2015; Hayder et al., 2018).

To date, most imprinted miRNA genes are generated from three evolutionarily different chromosomal domains, denoted hereafter as C19MC, C14MC, and C2MC (also known as *Sfmbt2* miRNA cluster). C19MC and C2MC are only expressed from the paternally inherited chromosome, while the expression of C14MC—embedded within the imprinted Dlk1-Dio3 domain—is restricted to its maternal allele (Figures 2B,C). Besides their mono-allelic expression, these peculiar miRNA genes share common features. First, they are organized as large repetitive arrays that spread over ~50–250 kb-long genomic regions containing dozens of miRNA genes, with some of them sharing sequence homology (Figure 2B). Second, they show tissue–specific expression, with marked or exclusive expression in placenta. The C14MC cluster is also highly expressed in the adult brain. Although little is known about the mechanisms underlying their spatiotemporal expression, imprinted miRNAs are believed to be co-expressed as, and processed from, a single (or few) primary transcripts. Third, they represent evolutionary novelties: C14MC is only found in eutherians, while C19MC and C2MC are restricted to, respectively, primates and rodents (Figure 2C).

Here, we overview the complex “ménage à trois” that evolutionarily and/or functionally links placentation, genomic imprinting and miRNAs. We argue that recently-evolved, imprinted miRNA gene clusters may have contributed to the phenotypic variability of the placenta, possibly by optimizing fetal-maternal exchanges within each species. Additional information regarding the biology of imprinted miRNA gene clusters, particularly in the regulation of brain functions, can be found elsewhere (Labialle et al., 2012; Labialle and Cavaille, 2011; Girardot et al., 2012; Benetatos et al., 2013; Winter, 2015; Marty and Cavaille, 2019).

## The Primate-Specific, Paternally-Expressed C19MC miRNA Genes

The Chromosome 19 MicroRNA Cluster (C19MC) at human Chr19q13 was first described by Bentwich et al. (2005). This primate-specific cluster is composed of 46 miRNA genes and occupies a ~100 kb-long region upstream of the miR-371/miR-373 cluster, the human ortholog of the ES cell-specific miR-290/295 cluster (Figure 2C). Many, if not all, C19MC miRNAs appear to be processed from introns of a poorly characterized transcript (named C19MC-HG) composed of many repeated non-coding exons (Bortolin-Cavaille et al., 2009). C19MC is highly enriched in evolutionary old Alu elements (AluJ and AluS) that very likely contributed to the evolution and growth of C19MC, possibly during an early wave of Alu expansion (Zhang et al., 2008; Lehnert et al., 2009).

**Figure 3:**
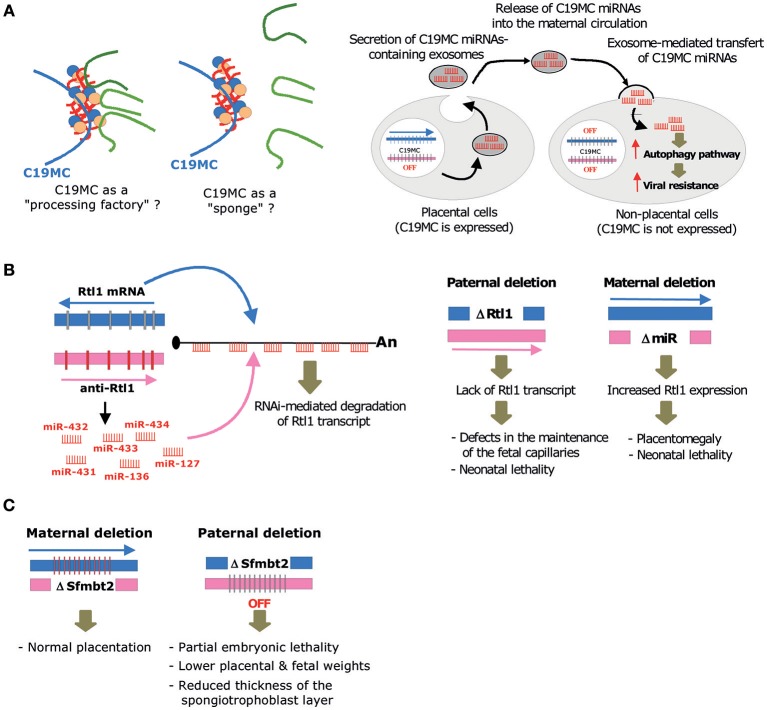
Molecular, cellular and biological, functions mediated by imprinted miRNA gene clusters. **(A)** Left: By concentrating locally the Microprocessor, C19MC may act as a “processing factory” that facilitates the processing of other pri-miRNAs generated elsewhere in the genome. On the contrary, it may also function as a “sponge” that sequesters the Microprocessor, thus limiting the processing of some pri-miRNAs. Right: C19MC miRNAs packaged into exosomes are taken up by non-placental cells where they elicit an antiviral response by promoting autophagy pathways. **(B)** Left: RNAi-mediated trans-interaction at the imprinted Rtl1/Peg11 locus. The maternally expressed anti-Rtl1 transcript (pri-miRNA) generates six miRNAs (miR-431, miR-433, miR-127, miR-434, miR-432, miR-136) that have fully complementary sequences to the paternally expressed Rtl1 mRNA, hence leading to its cleavage through RNA interference. Right: Main phenotypes observed upon paternal or maternal deletion at the Rtl1/miR-127-136 gene locus, as described in Sekita et al. (2008) **(C)** Main phenotypes observed upon maternal or paternal deletion at C2MC, as described in Inoue et al. (2017).

Under physiological conditions, C19MC is predominantly detected in placenta, as well as in undifferentiated embryonic stem (ES) cells and germ cells (Sadovsky et al., 2015). Of note, amplification of C19MC is also frequently observed in aggressive neuro-ectodermal tumors, where its ectopic expression triggers an embryonic expression program that promotes tumor formation in the infant brain (Li et al., 2009; Pfister et al., 2009; Kleinman et al., 2014). Remarkably, C19MC miRNAs are among the most abundant miRNAs expressed in human trophoblastic cells, at least in term placenta (Donker et al., 2012). C19MC is only active on the paternally inherited allele and its expression is driven by a Polymerase-II promoter region, mapping ~17 kb away from the first exon. This promoter overlaps a differentially methylated region (DMR) that acquires DNA methylation in the oocyte (Noguer-Dance et al., 2010). This DMR may therefore function as an imprinting control region (ICR) that dictates monoallelic expression not only at C19MC, but also possibly at the flanking maternally expressed ZNF331 gene (Noguer-Dance et al., 2010) and the paternally expressed transcript produced in the antisense orientation relative to the miR-371/miR-373 cluster (Stelzer et al., 2013). Surprisingly, C19MC-HG transcripts form large “RNA clouds” at transcription sites where they attract huge amounts of the Drosha-DGCR8 (Microprocessor) complex involved in the first nuclear step of pri-miRNA processing (Bellemer et al., 2012). The functional significance of these rather unexpected observations is unknown. As speculated in Figure 3A-left, C19MC may facilitate the processing of other pri-miRNAs generated elsewhere in the genome, as a result of the increased local concentration of the Microprocessor. Such “pri-miRNA factory” implies that the compartmentalization of Microprocessor at C19MC imposes long range interactions, whereby chromosomal regions harboring active miRNA genes relocate close to C19MC. Alternatively, C19MC may function as a “sponge” and negatively impact on pri-miRNA processing by reducing the availability of the nuclear pool of Microprocessor.

### C19MC Controls Migration and Invasion of Human Trophoblasts

Several studies in human choriocarcinoma or primary cultures of trophoblasts support the involvement of one or several members of C19MC in the physiology of trophoblasts (Table 1). A-case-in-point are members of the miR-515 family, which are significantly down-regulated during differentiation of primary cultured cytotrophoblasts into syncytiotrophoblasts. Conversely, miR-515-5p overexpression has been shown to inhibit differentiation of syncytiotrophoblasts. These miRNAs may target hCYP19A1/aromatase P450, glial cells missing 1 (GCM1) and frizzled 5 (FZD5) (Zhang et al., 2016). Furthermore, the forced expression of the entire C19MC in HTR-8/SVneo, an extravillous human trophoblast-derived cell line that normally does not express C19MC, attenuates cell migration without impacting on proliferation or apoptotic pathways (Xie et al., 2014). In this context, miR-519d was proposed to play a pivotal role by targeting CXCL6, NR4A2, and FOXL2 transcripts (Xie et al., 2014; Xie and Sadovsky, 2016). The ectopic expression of additional C19MC-derived miRNAs also modulates migration and invasion of extra-villous trophoblasts, notably via the action of miR-519d-3p (Ding et al., 2015), miR-520g (Jiang et al., 2017), miR-517a/b (Anton et al., 2015) and miR-520c-3p (Takahashi et al., 2017). A role of C19MC in placenta metabolism, presumably in the adaptation to hypoxia, is also emerging. Hypoxic stress is associated with reduced levels of miR-520c-3p while expression levels of the other C19MC miRNAs remain unchanged (Donker et al., 2012). Moreover, treating extra-villous trophoblasts with the hypoxia mimetic Cobalt Chloride also increased expression levels of miR-517a/b (Anton et al., 2015). Finally, an involvement of C19MC in the maintenance of the undifferentiated state of embryonic stem (ES) cells was also inferred. Indeed, 16 out of 46 C19MC miRNAs display the “AAGUGC” seed sequence found in miR-302, an ES cell-specific miRNA that promotes “stemness” [(Nguyen P. N. et al., 2017; Nguyen P. N. N. et al., 2017) and references therein]. Although C19MC expression correlates with the undifferentiated state of ES cells, there are no genetic evidence showing that endogenously-expressed C19MC miRNAs play a direct role in the self-renewal or growth of human ES cells. Of relevance, C19MC expression is higher in trophoblast cells as compared to ES cells (~10–10,000 fold differences) and, as such, high C19MC expression is now considered as one of the most robust markers to define trophoblastic cells (Lee et al., 2016). High C19MC expression was also found in some recently characterized human trophoblast stem cells (Okae et al., 2018). It is therefore tempting to speculate that some C19MC miRNAs may control stem-like traits of trophoblast stem cells, perhaps via the same regulatory pathways orchestrated by the miR-302/miR-367 cluster (Zhang et al., 2015).

**Table 1:** Physiological roles and predicted mRNAs targeted by placentally expressed, imprinted miRNA genes.

**Clusters**	**miRNAs**	**Models**	**Predicted targets**	**Placental functions**	**References**
C19MC	miR-512-3p	Human cells	PPP3R1	?	Kurashina et al., 2014
C19MC	miR-518c	Human cells	HSD17B1	?	Ishibashi et al., 2012
C19MC	miR-512-3p, miR-516b-5p , miR-517-3p	Human cells	?	Cell-to-cell communication (antiviral properties)	Delorme-Axford et al., 2013
C19MC	miR-517a-3	Human cells Maternal blood	PRKG1	Cell-to-cell communication (maternal natural killer cells)	Kambe et al., 2014
C19MC	miR-515-5p, miR-519e-5p, miR-519c-3p, miR-518f	Human cells	hCYP19A1 GCM1, FZD5	Syncytiotrophoblast differentiation (cell fusion)	Zhang et al., 2016
C19MC	miR-520c-3p	Human cells	?	Response to hypoxia ?	Donker et al., 2012
C19MC	miR-517a/b	Human cells	?	Response to hypoxia ?	Anton et al., 2015
C19MC	miR-519d	Human cells	CXCL6, NR4A2, FOXL2	Trophoblast migration	Xie et al., 2014; Xie and Sadovsky, 2016
C19MC	miR-519-3p	Human cells	MMP-2	Trophoblast migration and invasion	Ding et al., 2015
C19MC	miR-520g	Human cells	MMP-2	Trophoblast migration and invasion	Jiang et al., 2017
C19MC	miR-520c-3p	Human cells	CD44	Trophoblast invasion	Takahashi et al., 2017
C14MC	miR-127/miR136 cluster	KO mouse model	*Rtl1*	Placental development (Labyrinthe layer)	Sekita et al., 2008, Ito et al., 2015
C14MC	miR-376c	Human cells	ALK5, ALK7	Trophoblast proliferation, invasion, migration	Fu et al., 2013b
C14MC	miR-378-5p	Human cells	NODAL	Trophoblast proliferation, invasion, migration	Luo et al., 2012
C14MC	miR-378a	Human cells	CCNG2	Trophoblast differentiation	Nadeem et al., 2014
C14MC	miR-377	Human cells	?	Trophoblast proliferation	Farrokhnia et al., 2014
C2MC	miR6669f-3P, miR-466f-3p, miR6297a	Mouse cells	*Lats2, Dedd2*	Pro-survival functions ?	Zheng et al., 2011
C2MC	C2MC	KO mouse model	*Gkn2, Car8, Dlx4, Fndc1, Bmper, Fst, Sh3d2l, Rgs4, Fgfrl1*	Placental development (spongiotrophoblast layer)	Inoue et al., 2017

### C19MC and Cell-to-Cell Communications During Pregnancy

Another fascinating finding concerning C19MC resides in its role in resistance to viral infection, as demonstrated by the groups of Sadovsky and Coyne (Delorme-Axford et al., 2013). Indeed, term primary human trophoblasts, expressing high amount of C19MC, are resistant to infection by a various panel of DNA and RNA viruses including human cytomegalovirus (CMV), vesicular stomatitis virus (VSV), herpes simplex virus-1, vaccinia virus, poliovirus, and others (Delorme-Axford et al., 2013; Bayer et al., 2015, 2018). Introduction of C19MC through the use of a bacterial artificial chromosome in non-placental cells, permissive for viral infections, rendered them resistant to VSV infection. These antiviral properties were also recapitulated when three C19MC-encoded miRNAs—miR-517-3p, miR-516-5p, and miR-512-3p—were overexpressed as miRNA mimics. Strikingly, protection from VSV infection was also conferred by conditioned culture media, or even isolated exosomes, in which C19MC miRNAs are highly represented (Luo et al., 2009; Donker et al., 2012; Ouyang et al., 2016). Three major forms of extracellular vesicles can be secreted by cells: apoptotic blebs (1–5 μm), microvesicles (0.1–1 μm) and exosomes (40–150 nm) which all display variable biogenesis, composition and content (van Niel et al., 2018). Although these different types of trophoblastic extracellular vesicles contain a comparable repertoire of miRNAs, their phospholipid and protein contents differ. Among them, exosomes have been shown to confer the higher antiviral properties in non-trophoblastic cells (Ouyang et al., 2016).

Taken together, it has been proposed that C19MC miRNAs are packaged into exosomes, delivered into the bloodstream and incorporated by recipient cells, of either maternal or fetal origin, where they elicit an antiviral response by promoting autophagy pathways (Delorme-Axford et al., 2013; Figure 3A-right). The precise molecular and cellular bases of C19MC-mediated antiviral effects are yet to be determined. It is known, however, that C19MC expression is not modified upon viral infection (Dumont et al., 2017) and that the antiviral properties of C19MC do not depend on type III interferon signaling (Bayer et al., 2018). Of note, C19MC-mediated paracrine effects are very likely virus-specific, because they do not protect, and even potentiate, CMV infection (Delorme-Axford et al., 2013). Other studies also demonstrated an involvement of exosomal C19MC miRNAs in cell-to-cell communication, notably between placenta and immune cells (Kambe et al., 2014; Ishida et al., 2015). *In vitro*, miR-517a-3 secreted from human choriocarcinoma (BeWo) cells can be transferred into the immortalized T lymphocyte (Jurkat) cells, where it downregulates the expression of endogenous PRKG1 mRNA. Even more remarkably, miR-517a-3, as well as miR-518b, can also be detected in natural killer cells from the blood of pregnant women, particularly in the third trimester. Their levels rapidly decline after delivery, further arguing that exosome-mediated delivery of C19MC in immune cells occurs during pregnancy. Yet, their putative roles in immunity pathways remain to be unveiled (Ishida et al., 2015). Altogether, these data lend experimental support to the concept that C19MC miRNA-containing exosomes mount cellular responses to protect fetus from maternal immune activation and viral infection (Bullerdiek and Flor, 2012; Mouillet et al., 2014; Tong et al., 2018).

## The Eutherian-Specific, Maternally-Expressed C14MC miRNA Genes

The eutherian-specific, imprinted Dlk1-Dio3 domain plays essential roles in prenatal growth, placentation, skeletal and muscular development, postnatal metabolism, and brain functions (da Rocha et al., 2008; Edwards et al., 2008). This ~1 Mb genomic interval is located at human 14q32 (distal mouse chromosome 12) and comprises three paternally expressed, protein-coding genes: Delta-like homolog 1 (*Dlk1*), Retrotransposon-like 1 (*Rtl1*), and Type III iodothyronine deiodinase (*Dio3*). It also generates a complex set of maternally expressed non-coding RNA transcripts including Meg3 (previously named Gtl2), an antisense transcript to Rtl1 as well as numerous small RNAs belonging to the SNORD and miRNA families. In humans, miRNAs are collectively named C14MC (Chromosome 14 MicroRNA Cluster) and most of them are grouped into two genomic regions: the miR-127/miR-136 cluster and the miR-379-miR-410 cluster located upstream and downstream from the SNORD gene cluster, respectively (Figure 2C). During mouse development, miRNAs at the Dlk1-Dio3 domain are widely expressed in the embryo, the placenta and also to some extent in many tissues in the perinatal period. In adulthood, their expression becomes mostly restricted to the brain (Seitz et al., 2004; Labialle et al., 2014).

### The Mouse miR-127/miR-136 Cluster Regulates Fetal Capillaries in the Labyrinth Zone

The paternally expressed Rtl1 gene (also known as Peg11 or SIRH2) derives from Ty3/Gypsy type retrotransposon that has evolved a conserved open reading frame lacking long terminal repeats (Kaneko-Ishino and Ishino, 2012). Interestingly, Rtl1 retrotransposed into the Dlk1-Dio3 region before the split of the eutherians and metatherians, but it only became exapted in eutherians (Edwards et al., 2008; Kaneko-Ishino and Ishino, 2010). Remarkably, Rtl1 gene is associated with a maternally-expressed antisense transcript (anti-Rtl1) which also serves as the primary transcript for the miR-127/miR-136 cluster. In the mouse, *anti-Rtl1* generates six miRNAs: miR-431, miR-433, miR-127, miR-434, miR-432, miR-136. Due to their antisense genomic organization, these miRNAs are fully complementary to sequences of *Rtl1* mRNA (Seitz et al., 2003) and they are thus able to cleave *Rtl1* mRNA through RNA interference (Davis et al., 2005; Figure 3B-left).

In order to decipher the biological roles of the *Rtl1* gene and its associated antisense transcript in placentation, Sekita et al have generated a knockout mouse bearing a deletion encompassing the Gag and Pol-like domain of *Rtl1* (Sekita et al., 2008). A paternal deletion (denoted hereafter ΔPat) resulted in late fetal and neonatal lethality accompanied by pre- and post-natal growth retardation of the embryo and the placenta. Upon maternal transmission (denoted hereafter ΔMat), neonatal lethality was also observed, with pups at P1 of normal weight but presenting a 156% placentomegaly. Thus, loss or overexpression of *Rtl1* (~2.5–3 fold change expected due to the lack of miRNA expression) affects the development of the embryo and the placenta (Figure 3B-right). Closer examination showed that ΔPat and ΔMat placentas present reciprocal abnormalities mostly detected in the labyrinth zone, where fetal-maternal interactions occur. ΔPat placentas displayed a splitting of the basement membrane, layer III trophoblasts with numerous lysosomes and a clogging of the fetal capillaries. Further staining using the CD31 marker revealed a phagocytic reaction to endothelial cells by trophoblast cells. These abnormalities impaired maternal/fetal transport: nutrition transfer assays indicated that passive permeability of insulin was severely diminished in ΔPat whereas active transport remained unaffected. Conversely, the placentomegaly in ΔMat was associated with enlargement of the inner spaces of fetal capillaries, with layer III trophoblasts presenting large vacuoles corresponding to starving cells. Finally, cell staining indicated that the Rtl1 protein was localized around the nuclei of endothelial cells, suggesting a role of this protein in the maintenance of the fetal-maternal interface. A more recent work by Kitazawa et al. has investigated these phenotypes in more details (Kitazawa et al., 2017).

Ito and colleagues also generated another knockout mouse carrying a smaller deletion that only removed miR-127 on the maternal allele (ΔMat_miR-127), while generating a non-sense mutation inducing a premature translation termination of *Rtl1* on the paternal allele (ΔPat_Rtl1) (Ito et al., 2015). Interestingly, ΔMat_miR-127 mice recapitulate the placentomegaly observed in the above-mentioned study, although to a lesser extent (~111–118% as opposed to 158%). No lethality and no major effect on fetal weight were noticed. As expected, the expression of all *Rtl1* isoforms was increased in both miR-127-deficient embryos and placentas. Thus, miR-127 contributes significantly in the silencing of *Rtl1* mRNA. This observation is consistent with previous reports in mouse embryonic stem cells showing that miR-127 is the main contributor of Rtl1 silencing (Ciaudo et al., 2009). The morphological study of the placenta revealed an expanded labyrinth zone, while the other layers remained unchanged. More specifically, the fetal capillaries and the labyrinthine trophoblasts were significantly increased. No major change was noticed regarding the thickness of the interhemal trophoblast membrane. ΔPat_Rtl1 placentas had an opposite phenotype, with consistent alterations of the maternal blood space and fetal capillaries. Of note, the theoretical and specific diffusion capacity of ΔMat_miR-127 placenta is higher than in wild-type mice but does not result in overgrowth, while their diminution in ΔPat_Rtl1 placenta may contribute to growth retardation. Altogether, these data point to a key role of the paternally expressed *Rtl1* gene in the growth of fetal capillaries of the labyrinth layer, whereas the maternally expressed miR-127/miR136 cluster, and particularly miR-127, antagonizes these growth-promoting effects (Sekita et al., 2008; Ito et al., 2015).

To date, the function of the other miRNAs at the Dlk1-Dio3 in placenta is less understood. A maternal transmission of a deletion encompassing the entire miR-379/miR-410 cluster is associated with partially penetrant neonatal lethality (~30%), probably due to impaired metabolic pathways resulting from defects in the activation of the neonatal hepatic gene expression program (Labialle et al., 2014). An involvement of the miR-379/miR-410 cluster in regulating placental function has never been directly addressed so far. Of note, *in vitro* studies with the human trophoblast cell line *HTR8*/*SVneo* showed that gain-of-function of one of the members of the miR-379/miR-410 cluster, namely miR-376c, promotes proliferation, migration and invasion (Fu et al., 2013b). In addition, it also induces placental explant outgrowth by targeting activin receptor-like kinase 5 (ALK5), a type I receptor for transforming growth factor-β and ALK7, a type I receptor for Nodal. Finally, a role in the control of trophoblast proliferation has also been ascribed for human miR-377, another member of C14MC, which is upregulated in term placenta vs. early placenta (Farrokhnia et al., 2014).

## The Rodent-Specific, Paternally-Expressed C2MC miRNA Genes

*Sfmbt2* (Sex combs on the midleg with four MBT domains) was identified by a screen aiming at identifying new imprinted genes involved in trophoblast development (Kuzmin et al., 2008). This gene is conserved amongst eutherians and encodes a protein of the Polycomb family. In the mouse, *Sfmbt2* is expressed from the paternal allele in early embryos and extraembryonic tissues, but it lacks imprinted expression in somatic tissues after E7.5 (Kuzmin et al., 2008). *Sfmbt2* is located in a ~4 Mb gene desert and the most proximal genes do not seem to be imprinted (Wang et al., 2011). The intron 10 of mouse *Sfmbt2* gene contains a large cluster of miRNA genes formed by tandem duplication of a basic unit composed of repeated B1 retrotransposons (Lehnert et al., 2011). This is the *Sfmbt2* miRNA cluster or Chromosome 2 MicroRNA Cluster (C2MC), which is only found in rodents where its expression is restricted to the paternal allele, similar to its host-gene (Inoue et al., 2017).

### *Sfmbt2* miRNAs Control the Development of the Spongiotrophoblast Layer

An initial study demonstrated that *Sfmbt2* is a trophoblast regulator. Indeed, paternal inheritance of a gene trap null allele is lethal before E12.5, due to the lack of all trophoblast cell types and reduced placenta size. Moreover, early embryos transduced by lentiviral vectors expressing anti-*Sfmbt2* shRNAs display reduced stem cell derivation as compared to wild-type (Miri et al., 2013). These experiments did not distinguish the respective contributions of *Sfmbt2* itself from the intron-encoded C2MC miRNAs. Using CRISPR/Cas9 technology, Inoue and colleagues have recently performed a targeted deletion of a 53 kb region encompassing the entire miRNA cluster, without affecting the expression of its host gene (Inoue et al., 2017). Homozygous mutant mice or paternal transmission of the deletion both result in partial embryonic lethality. However, mutants that survive generally reach adulthood. Conceptuses reaching term have lower body weight and display placental defects. Placenta is smaller and the global organization of the placental layers are altered, especially for the spongiotrophoblast, which is thinner (Figure 3C). Disruption of the parietal yolk sac (due to a thinner Reichert's membrane and a decreased number of endoderm cells) creates a gap with the placenta. Lack of C2MC has a strong impact on the whole transcriptome. Indeed, micro-arrays analyses performed in C2MC-deficient placentas at E8.5 and E11.5 identified 7,839 and 3118 up-regulated probes, respectively. Subsequent *in silico* analyses showed that 15 putative C2MC target genes were upregulated in both developing stages, with 9 out of 15 being further validated by RT-qPCR. These include tumor suppressor genes, apoptosis inducers and differentiation/patterning-regulating genes, thus suggesting a role for C2MC miRNAs in the regulation of proliferation/apoptosis and migration/invasion processes during placentation (Inoue et al., 2017).

## Imprinted miRNAs in Pathological Conditions of Pregnancy

C14MC and C19MC miRNAs are regulated in a spatiotemporal manner during human placental development. C19MC expression globally increases during pregnancy, from the first to third trimester (Luo et al., 2009; Donker et al., 2012; Morales-Prieto et al., 2012). However, expression levels may vary from one miRNA to another. For example, the expression of miR-518b, miR-519a, miR-520a, miR-520c, miR-520h, miR-526a is higher at early gestation stages than in late placentas (Hromadnikova, 2012; Gu et al., 2013). C19MC miRNAs are also regulated in a spatial manner, with expression levels of miR-517-3p, miR-518b, miR-519d, miR-520g, miR515-5p, and miR-1323 being higher in villous trophoblasts than in extra-villous trophoblasts (Xie et al., 2014). Of note, C19MC expression was also reported in placenta-derived mesenchymal stromal cells, thus indicating that regulatory functions mediated by C19MC may not be limited to trophoblasts (Flor et al., 2012). In contrast to C19MC, C14MC expression globally decreases in the placenta, from the first to the third trimester of pregnancy (Morales-Prieto et al., 2012). Notably, 11 miRNAs from C14MC are more expressed in the first trimester than in the third trimester placenta (Gu et al., 2013). Moreover, although a global decrease of C14MC expression was also reported in rhesus macaque placenta, C19MC expression was not increased in term placenta, thus suggesting species-specific differences among primates (Schmidt et al., 2018). Moreover, aberrant expression of miRNAs in general, and of C19MC miRNAs in particular, is frequently detected in various pregnancy complications including preeclampsia (PE), intrauterine growth restriction (IUGR) or preterm birth. Below are quoted a few studies. Additional information regarding differential expression of C19MC or C14MC in placental health can be found in more detailed review articles (Fu et al., 2013a; Morales-Prieto et al., 2013; Escudero et al., 2016; Sheikh et al., 2016; Cai et al., 2017).

PE affects around 3–8% of pregnancies worldwide (Sibai et al., 2005). The first stage of PE is a placentation defect, with impaired cytotrophoblast differentiation during their invasion into the spiral uterine arteries. This elicits a decrease of placental size and a restriction of utero-placental blood flow. The resulting ischemia/hypoxia leads to apoptosis and necrosis, together with release of cellular debris by the trophoblasts. These changes lead to the onset of the second stage of PE that occurs after 20 weeks of gestation. This stage is characterized by hypertension, proteinuria, thrombocytopenia, renal insufficiency, impaired liver functions amongst others for the mother, and poor growth and prematurity or even perinatal mortality for the baby. After a first work published in 2007 (Pineles et al., 2007), an ever increasing number of miRNA profiling studies have identified differentially expressed imprinted miRNAs in the placenta and circulation of preeclamptic women, as compared to normal pregnancy. Decrease of some C14MC- or C19MC-derived miRNAs (Enquobahrie et al., 2011) were described in the placental tissues, with the identification of miR-519a as a potential marker of severe PE (Hromadnikova et al., 2015). Another study also reported increased or decreased C14MC and C19MC miRNAs in the placenta (Zhu et al., 2009). Analyses of serum plasma from mothers revealed a global increase of circulating miRNAs, with some C19MC-derived miRNAs being overexpressed (Miura et al., 2015). Up-regulation of miR-517-5p, miR-518b, and miR-520h were associated with a risk of developing PE, especially miR-517-5p which was proposed as a reliable predictive biomarker (Hromadnikova et al., 2017b). A systematic study comparing miRNA levels in placenta and blood plasma from pregnant women at early or late onset of PE showed that miR-423-5p, 519a-3p, and-629-5p were less expressed in the placenta, yet they were enriched in the blood plasma, identifying miR-423-5p as another potential biomarker for early stage of PE (Timofeeva et al., 2018). Finally, miR-520g and miR-515 were also reported to display elevated levels in PE (Zhang et al., 2016; Jiang et al., 2017). Although the above-mentioned studies lack consistency, a recent comprehensive literature review identified two members of C19MC, miR-518b, and miR-517c, as the most frequently differentially expressed miRNAs (Top10) across published reports of PE (Sheikh et al., 2016).

Imprinted miRNAs are also dysregulated during IUGR (Mouillet et al., 2010; Fu et al., 2013a). This disorder is characterized by a failure for the fetus to grow and reach its normal weight with a significant risk of perinatal morbidity and mortality (Resnik, 2002). In placental tissues of IUGR, the expression of several C19MC-derived miRNAs (miR-518b, miR-1323, miR-516b, miR-515-5p, miR-520h, miR-519d, and miR-526b) was decreased (Higashijima et al., 2013; Wang et al., 2014), while another study showed that miR-519a expression was increased (Wang et al., 2014). However, further studies did not detect any modification of C19MC-derived miRNAs in plasma samples from mothers with fetuses presenting an IUGR (Hromadnikova et al., 2013, 2017b), perhaps reflecting the fact that plasma levels of C19MC-miRNAs do not reflect their placental expression.

Few studies have investigated the correlation between preterm birth and dysregulations of miRNA expression (Elovitz et al., 2015; Manokhina et al., 2015; Hromadnikova et al., 2017a; Winger et al., 2017). Among them, one study showed that upregulation of C19MC miRNAs in placental tissues (miR-515-5p, miR-516-5p, miR-518b, miR-518f-5p, miR-519a, miR-519e-5p, miR-520a-5p, miR-520h, and miR-526b-5p) is a characteristic phenomenon of preterm birth (Hromadnikova et al., 2017a). However, other analyses of the miRNA content of extracellular vesicles showed that expression level of C19MC, as well as that of C14MC, tended to decrease in preterm labor in pregnant women (Fallen et al., 2018). Finally, it has recently been suggested that changes in the expression of C19MC and C14MC miRNAs, considered as a group and as measured in maternal plasma, might be predictive of infant head circumference and length of gestation, respectively (Wommack et al., 2018).

## Conclusions—Open Questions

**Figure 4:**
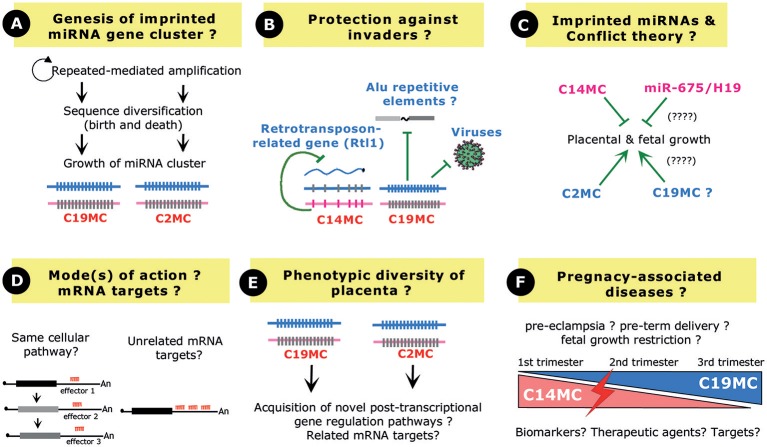
Perspective and Open questions. **(A)** Evolution of a repeated array of miRNA genes. Novel miRNA genes may have arisen from amplification of a single (or few) ancestral miRNA gene(s) followed by sequence diversification through a birth and death process. Note that the growth of C19MC and C2MC has been facilitated by interspersed repeated elements (Alu elements and repeated B1 retrotransposons, respectively). **(B)** Imprinted miRNA gene clusters may confer protection against “genomic invaders,” by inhibiting endogenous retrotransposons, interspersed Alu repeats or viruses. **(C)** In the framework of the parent–offspring conflict theory, the paternally expressed C2MC (and also possibly C19MC?) promotes placental growth to increase the fitness of the fetus while, on the contrary, the maternally expressed C14MC limits placental growth. The maternally expressed, single copy miR-675 gene may also exert related restricted growth functions. **(D)** Imprinted miRNA gene clusters may target different mRNAs involved in the same biological pathway and/or could target the same 3′-UTR, very likely in a synergistic fashion. **(E)** Several miRNAs at C19MC and C2MC share the same “AAGUGC” seed region, leaving open the possibility that, through convergent evolution, they target the same set of mRNA targets in primates and rodents, respectively. **(F)** C14MC and C19MC miRNAs show opposite expression profiles in the maternal serum during pregnancy. Their expression is often found deregulated in pathological pregnancies such as pre-eclampsia, preterm birth or fetal growth restriction, thus opening the possibilities to use them as biomarkers, targets, or therapeutic agents.

C2MC, C14MC, and C19MC represent recent gene innovations. They have likely arisen from segmental duplication of a single (or a few) ancestral miRNA gene copy followed by sequence diversification through a “birth-and-death” process (Glazov et al., 2008; Zhang et al., 2008; Lehnert et al., 2009, 2011). Imprinted gene clusters can therefore be considered as “miRNA nurseries” from which novel miRNA species have rapidly evolved or are still even emerging (Figure 4A). Such a scenario may also imply that all miRNAs are not necessarily functional within a given cluster. In any case, lineage- or even species-specific miRNAs could then become integrated into pre-existing gene circuitries or dictate novel posttranscriptional gene regulation pathways. For instance, computational work showed that C2MC has a genome-wide impact on the mouse transcriptome, with predicted mRNA targets being under positive selection (Zheng et al., 2011). Evolutionary young miRNAs have the ability to confer phenotypic differences between organisms (Mor and Shomron, 2013) and, as such, imprinted miRNA gene clusters have very likely sculpted the variability in the morphology and function of the placenta among mammals.

Mouse genetics have further established that C2MC and C14MC (the miR-127/miR-136 cluster) play essential roles in the maintenance of the spongiotrophoblast and labyrinth layers, respectively (Sekita et al., 2008; Ito et al., 2015; Inoue et al., 2017). Regarding C19MC, a formal demonstration of its direct contribution to primate placentation at the organism level is still lacking. Nevertheless, *in vitro* studies as well as *in vivo* miRNA measurements in maternal blood (Dumont et al., 2017) provide evidence for their involvement in the migration and invasion of trophoblasts during early pregnancy. As nicely exemplified by C19MC, exosome-mediated delivery of imprinted miRNAs can also confer antiviral immunity to non-placental cells (Delorme-Axford et al., 2013). Furthermore, C19MC miRNAs may counteract the potential detrimental effects of Alu repeated elements (Lehnert et al., 2009). The miR-127/miR-136 cluster at C14MC represses a retrotransposon-like gene (Davis et al., 2005). Another intriguing theme is therefore emerging where imprinted miRNA gene clusters may also act as “guardians” in host defense mechanisms (Figure 4B), by limiting the activity of genomic or cellular parasites whether viruses, interspersed repeats or endogenous retrotransposons (Seitz et al., 2003; Lehnert et al., 2009; Delorme-Axford et al., 2013; Mouillet et al., 2014).

Beside the three above-mentioned imprinted arrays of repeated miRNA genes, there are also a few imprinted miRNAs produced by a single gene locus: the paternally expressed miR-335 gene at the human 7q32/imprinted MEST domain (Hiramuki et al., 2015), the paternally expressed miR-296 gene at the human 20q13/imprinted GNAS domain (Robson et al., 2012), the paternally-expressed miR-374-5p and miR-421-3p genes on mouse X chromosome (Kobayashi et al., 2013) and the maternally expressed miR-675 gene at the human 11p15.5/imprinted H19/Igf2 domain (Smits et al., 2008). So far, only miR-675 was proposed to play some roles in placentation. This highly conserved miRNA, also found in Marsupials, is embedded within exon 1 of the maternally expressed ncRNA H19 (Smits et al., 2008). In the developing placenta, the processing of miR-675 is inhibited by the RNA-binding protein HuR except at late stage of gestation where growth of the placenta naturally ceases. Thus, H19 can be seen as a “reservoir” of miR-675 whose expression increases in the late gestation, particularly in the labyrinth zone (Keniry et al., 2012). Ectopic expression of miR-675 reduced the proliferation of the transfected cells and, remarkably, its knock-out in the mouse, at least as judged by the H19Δ3 mouse strain, results in placental overgrowth. Altogether, it has been surmised that the developmentally regulated release of miR-675 from H19 limits placental growth before birth, very likely by dampening the expressing of the growth-promoting insulin-like growth factor 1 receptor (Igf1r) (Keniry et al., 2012). Finally, *in vitro* studies also showed that miR-675 has growth-suppressive effects when overexpressed in the human choriocarcinoma JEG-3 cells (Gao et al., 2012).

Many theories have been proposed to explain the emergence of genomic imprinting in mammals (Sleutels and Barlow, 2002; Renfree et al., 2009; Kaneko-Ishino and Ishino, 2010; Keverne, 2014). One of the widely cited, but still subjected to a lively debate, is the parent–offspring conflict or kinship theory (Moore and Haig, 1991; Haig, 2004). Put in very simple terms, this theory states that paternally expressed genes have been selected to increase the fitness of the fetus, by increasing extraction of nutrients even at the expense of maternal resource. On the contrary, maternally expressed genes may have evolved to counteract such growth-promoting effects, by reducing the demand for nutrients. Along this framework, the placenta is obviously one of the most relevant tissues where such a “tug-of-war” may take place. Our current understanding of the functions of imprinted miRNA gene clusters in placenta fits relatively well within these theoretical considerations. The paternally expressed C2MC promotes placental growth, as evidenced by reduced size of the placenta in C2MC-deficient mice, while C14MC limits placental growth, as exemplified by placentomegaly in miR-127/miR-136-deficient mice (Figure 4C). Outstandingly, the maternally expressed miR-127/miR-136 cluster represses paternally expressed Rtl1 transcripts (Seitz et al., 2003), a finding reminiscent of another “emblematic molecular arm race” operating, at the protein levels, between the two reciprocally *Igf2* and *Igf2r* imprinted genes (Seitz et al., 2003; Davis et al., 2005). Indeed, Igf2r, whose gene is active on the maternal allele, targets for degradation the growth-enhancer Igf2 whose gene is expressed from the paternal allele (Wang et al., 1994). Altogether, it is tempting to speculate that some imprinted miRNA genes, like some imprinted protein-coding genes, may have evolved to balance maternal resource acquisition in eutherian species. The relationship between genomic imprinting and miRNA gene cluster is very likely even more complex and interlaced. Indeed, S*fmbt2* is biallelically expressed in humans, cows or even in rodents from the Peromyscine genus and, remarkably all these species lack miRNA cluster in their syntenic regions (Wang et al., 2011). Thus, one appealing hypothesis is that acquisition of genomic imprinting at the *Sfmbt2* gene, and also possibly at other small RNA gene clusters, could be causally linked to the presence of the repeated small RNA gene arrays. Upon their amplification, miRNA gene clusters could have been recognized as expansion of foreign DNA and, according to the host-defense hypothesis (Barlow, 1993), might have attracted silencing chromatin machineries in only one of the two parental germ lines [(Labialle and Cavaille, 2011; Wang et al., 2011) and references therein].

To the notable exception of the miR-127/miR-136 cluster-Rtl1 target gene pair, for which expected endonucleolytic cleavages were mapped *in vivo* (Davis et al., 2005), mRNA targets for imprinted miRNAs were mostly inferred from *in silico* predictions, anti-correlated miRNA-mRNA expression and/or overexpression studies of individual miRNA (Table 1). Although informative and widely used in miRNA field, these approaches do not provide a formal demonstration of the biological importance of the tested miRNA. Moreover, and in our point of view, the concerted spatiotemporal expression of clustered imprinted miRNA genes might also be taken into consideration. In other words, we believe that their co-expression pattern matters. One can reasonably consider that clustered miRNAs function as “rheostats” to buffer protein levels involved in the same cellular pathways. Alternatively, they could also bind simultaneously to the same 3′-UTR and synergistically act as “switches” to fully dampen protein output. These two possibilities are obviously not mutually exclusive and could operate in a cell-context or species-specific manner (Figure 4D). Of primary interest, several miRNAs at C19MC (e.g., miR-520s) and CM2C (e.g., miR-467s) contain the similar “AAGUGC” seed which is also found in members of the miR-17/92 cluster (oncomiR1), the miR-371/miR-373 cluster or the ES-cell specific miR-302/367 cluster (Noguer-Dance et al., 2010) and references therein). Recent findings in cancer research point to a role of this seed sequence in reinforcing the proliferative cellular program in a wide range of cancers, by repressing tumor suppressor genes such as PTEN, RBL2, LATS2, or CDKN1A (Zhou et al., 2017). Moreover, bio-informatic analyses showed that predicted mRNA targets for C2MC are enriched in pathways that regulate growth, including *Lats2* mRNA (Zheng et al., 2011). The question then arises as whether some miRNAs generated at C2MC and C19MC may have related mRNA targets (or families thereof), perhaps underlying similar functions, i.e., proliferation and survival (Figure 4E).

Finally, the possibility to monitor miRNA levels in the serum of pregnant women in pathologic pregnancies has boosted a new field of research, focused on miRNA-based diagnostic and therapeutic tools. Indeed, although the direct cause and effect relationship between abnormal miRNA expression and the physiopathology of pregnancy diseases of multifactorial origin is far from being demonstrated, many efforts are currently being developed to identify non-invasive miRNA biomarkers for placental dysfunctions (Zhao et al., 2013). Due to their high expression levels in both placenta and mother sera, imprinted miRNAs, and particularly C19MC, have stimulated great interest, either as biomarkers, targets, or therapeutic agents (Figure 4F). One must admit, however, that many discrepancies still persist in the available literature, very likely due to the processing samples, cohort characteristics, gestational age, profiling technologies, and computer analyses. To date, these issues complicate the identification of a reliable biomarker for pregnancy outcome (Pritchard et al., 2012; Fu et al., 2013a; Moldovan et al., 2014; Mouillet et al., 2015; Tsochandaridis et al., 2015; Sheikh et al., 2016; Cai et al., 2017).

In conclusion, despite recent advances in our understanding of the biology of placentally expressed, imprinted miRNA gene clusters, there remain, to date, many persisting open questions. Indeed, the full repertoire of their physiologically relevant mRNA targets has yet to be precisely characterized at the whole organism level. In that respect, the use of CRISPR/Cas9 methods should now greatly facilitate the targeted disruption of putative miRNA binding sites onto endogenously-expressed mRNAs. Hopefully, this will provide novel insights into the gene regulatory pathways underlying genotype/phenotype relationships observed in knockout mice or cellular models. Our understanding of the biology of RNA-containing extracellular vesicles in general, and that of C19MC in particular, remains also sketchy (Mateescu et al., 2017). There are still many unresolved questions regarding how C19MC miRNAs are packaged within exosomes, how C19MC miRNA-containing exosomes reach specifically their target cells and, above all, how C19MC miRNAs impact on gene network in recipient cells, especially given that only tiny amounts of miRNAs are actually expected to be packaged in exosomes (Chevillet et al., 2014). More sophisticated studies are now required to fully appreciate the physiological impact of these recently-evolved, epigenetically-regulated miRNA gene clusters in placentation, cell-to-cell communication and also possibly during pathological pregnancies.

## Author Contributions

All authors listed have made a substantial, direct and intellectual contribution to the work, and approved it for publication.

### Conflict of Interest Statement

The authors declare that the research was conducted in the absence of any commercial or financial relationships that could be construed as a potential conflict of interest.
